# List of Cases Treated in the Surgical Wards of the Pennsylvania Hospital, and Discharged between November 28th and December 12th

**Published:** 1838-12-19

**Authors:** Henry H. Smith

**Affiliations:** Resident Surgeon


### CLINICAL REPORTS.

### PENNSYLVANIA HOSPITAL.

*List of Cases treated in the Surgical Wards of the Pennsylvania Hospital, and discharged between November 28th and December 12th.* Dr. T. Harris, Attending Surgeon.

[Reported by Henry H. Smith, M.D., Resident Surgeon.]

A case of contusion and lacerated wound of the foot, from a rail-road accident, was admitted September 6th. Sloughing took place, followed by a large and ill-conditioned sore: treated by rest, &c., and discharged cured, December 8th, ninety-three days after admission.

A case of fractured clavicle, near the humeral end, was admitted October 28th; dressed with the clavicle apparatus. The patient was afterwards attacked with rheumatism, treated accordingly, and discharged December 10th.

A case of simple fracture of the tibia, (about two inches above the ankle,) and of the upper third of the fibula, in an old drunkard, was admitted November 26th. The limb was placed in a fracture box, securely fastened to the bed, and laudanum, a blister, and stimulants, exhibited to meet symptoms of mania-a-potu, which appeared three hours after the man's entrance. He died November 29th, three days after admission.

A case of compound fracture of the leg, complicated with a compound dislocation of the ankle, in a man sixty-six years of age, caused by several cars passing over the limb, was admitted October 22d. The limb was amputated four hours after his entrance. The stump healed in forty days, but was afterwards attacked by erysipelas; treated accordingly, and the patient discharged December 10th.

A case of simple fracture of both bones of the leg, caused by a fall, was admitted September 14th. The man was of a bad constitution, was attacked with mania-a-potu shortly after entering the hospital, and, by his violent exertions, forced the bones through the integuments. A large slough afterwards occurred, and the limb was amputated below the knee, ten days after the man's admission. The stump afterwards sloughed, and the patient was not discharged until December 10th, eighty-seven days after the accident, and seventy-seven after the amputation.

A case of compound fracture of the condyles of the femur, (reported at length in number 24,) was admitted October 2d, the limb amputated, and the man discharged cured, December 12th, thirty-five days after the operation.

A case of a severe lacerated wound of both thumbs, caused by machinery, was admitted October 17th, and discharged cured, December 10th. Reported at length in this number.

A case of severe contusion of the hip and back, caused by a fall from the fourth story of a house, was admitted December 10th; treated by frictions and rest. Discharged at his own request, December 12th, two days after his entrance.

